# Gal-1 Expression Analysis in the GLIOCAT Multicenter Study: Role as a Prognostic Factor and an Immune-Suppressive Biomarker

**DOI:** 10.3390/cells12060843

**Published:** 2023-03-08

**Authors:** Neus Martínez-Bosch, Noelia Vilariño, Francesc Alameda, Sergi Mojal, Montserrat Arumí-Uria, Cristina Carrato, Iban Aldecoa, Teresa Ribalta, Noemí Vidal, Beatriz Bellosillo, Silvia Menéndez, Sonia Del Barco, Oscar Gallego, Estela Pineda, Raquel López-Martos, Ainhoa Hernández, Carlos Mesia, Anna Esteve-Codina, Nuria de la Iglesia, Carme Balañá, María Martínez-García, Pilar Navarro

**Affiliations:** 1 Cancer Research Program, Hospital del Mar Medical Research Institute (IMIM), Unidad Asociada IIBB-CSIC, 08003 Barcelona, Spain; 2 Medical Oncology Department, Hospital Duran i Reynals, Catalan Institute of Oncology, L’Hospitalet, 08908 Barcelona, Spain; 3 Department of Pathology, Hospital del Mar, 08003 Barcelona, Spain; 4 Institut de Recerca de l’Hospital de la Santa Creu i Sant Pau, Institut d’Investigacions Biomèdiques IIB-Sant Pau, 08025 Barcelona, Spain; 5 Department of Pathology, Hospital Germans Trias i Pujol, 08916 Badalona, Spain; 6 Department of Pathology, Center for Biomedical Diagnosis, Hospital Clinic de Barcelona, University of Barcelona, 08036 Barcelona, Spain; 7 Neurological Tissue Bank of the Biobank-Hospital Clinic-IDIBAPS (Instituto de Investigaciones Biomédicas August Pi i Sunyer), 08036 Barcelona, Spain; 8 Department of Pathology, Hospital Universitari de Bellvitge, L’Hospitalet, 08907 Barcelona, Spain; 9 Medical Oncology, Institut Catala d’Oncologia (ICO) Girona, Hospital Josep Trueta, 17007 Girona, Spain; 10 Department of Medical Oncology, Hospital de Sant Pau, 08036 Barcelona, Spain; 11 Department of Medical Oncology, Hospital Clínic Barcelona, Translational Genomics and Targeted Therapeutics in Solid Tumors, August Pi i Sunyer Biomedical Research Institute (IDIBAPS), 08036 Barcelona, Spain; 12 Badalona Applied Research Group in Oncology (B-ARGO Group), Institut Investigació Germans Trias i Pujol (IGTP), Institut Catalá d’Oncologia (ICO), 08916 Badalona, Spain; 13 Neuro-Oncology Unit and Medical Oncology Department, Institut Catala d’Oncologia (ICO), Institut de Investigació Bellvitge (IDIBELL), L’Hospitalet, 08908 Barcelona, Spain; 14 CNAG-CRG, Centre for Genomic Regulation, Barcelona Institute of Science and Technology (BIST), 08028 Barcelona, Spain; 15 IrsiCaixa AIDS Research Institute, Hospital Universitari Germans Trias i Pujol, 08916 Badalona, Spain; 16 Department of Medical Oncology, Hospital del Mar, 08003 Barcelona, Spain; 17 Cancer Research Program, Hospital del Mar Medical Research Institute (IMIM), 08003 Barcelona, Spain; 18 Departamento de Muerte y Proliferación Celular, Instituto de Investigaciones Biomédicas de Barcelona–Centro Superior de Investigaciones Científicas (IIBB-CSIC), 08036 Barcelona, Spain; 19 Institut d’Investigacions Biomèdiques August Pi Sunyer (IDIBAPS), 08036 Barcelona, Spain

**Keywords:** Galectin-1, glioblastoma, prognostic factor, *IDH-1*, mesenchymal molecular subtype, immune-suppression

## Abstract

Glioblastoma (GBM) is the most frequent primary malignant brain tumor and has a dismal prognosis. Unfortunately, despite the recent revolution of immune checkpoint inhibitors in many solid tumors, these have not shown a benefit in overall survival in GBM patients. Therefore, new potential treatment targets as well as diagnostic, prognostic, and/or predictive biomarkers are needed to improve outcomes in this population. The β-galactoside binding protein Galectin-1 (Gal-1) is a protein with a wide range of pro-tumor functions such as proliferation, invasion, angiogenesis, and immune suppression. Here, we evaluated Gal-1 expression by immunohistochemistry in a homogenously treated cohort of GBM (the GLIOCAT project) and correlated its expression with clinical and molecular data. We observed that Gal-1 is a negative prognostic factor in GBM. Interestingly, we observed higher levels of Gal-1 expression in the mesenchymal/classical subtypes compared to the less aggressive proneural subtype. We also observed a Gal-1 expression correlation with immune suppressive signatures of CD4 T-cells and macrophages, as well as with several GBM established biomarkers, including SHC1, PD-L1, PAX2, MEOX2, YKL-40, TCIRG1, YWHAG, OLIG2, SOX2, Ki-67, and SOX11. Moreover, Gal-1 levels were significantly lower in grade 4 *IDH-1* mutant astrocytomas, which have a better prognosis. Our results confirm the role of Gal-1 as a prognostic factor and also suggest its value as an immune-suppressive biomarker in GBM.

## 1. Introduction

Glioblastoma (GBM) is the most common primary tumor of the central nervous system (CNS) in adults, with a very dismal prognosis and a median overall survival of around 15 months after diagnosis [1,2]. Standard treatment includes maximal save resection followed by radiotherapy with concomitant and adjuvant temozolomide. Tumor-treating fields have shown additional modest survival benefits [3,4]. The isocitrate dehydrogenase 1 (*IDH-1*) mutation, although not frequent, confers a good prognosis in histological GBM tumors; for that reason, these tumors are now classified as astrocytomas grade 4 *IDH-1* mutant tumors in the new 2021 WHO classification [5,6]. Other indicators, such as the type of surgery, the patient’s age at diagnosis, and the Karnofsky Performance Status (KPS), are also known prognostic factors [7].

Recent advances in “omic” technologies have allowed for the establishment of molecular subtype classifications of GBM. The Cancer Genome Atlas (TCGA) and the Intrinsic Glioma Subtypes (IGS) classifications identified potential targets for specific populations within patients with GBM [8,9]. Recently, it has also been observed that immunohistochemistry (IHC) analysis, a technique that could be more easily incorporated into the diagnostic routine, can reflect molecular data [10,11]. Indeed, the importance of integrated diagnoses using molecular, histological, and IHC approaches has been incorporated into the new WHO classification of CNS tumors published in 2021 [6].

Galectins are a structurally related family of animal lectins defined by their affinity for β-galactoside structures through their carbohydrate-recognition domain (CRD). They function extracellularly by interacting with the cell surface and extracellular matrix glycoproteins, and intracellularly by interacting with cytoplasmic and even nuclear proteins [12,13]. One of the best-characterized members of the family is Galectin-1 (Gal-1). Gal-1 (*LGALS1*, ENS0000100097) is differentially expressed in normal versus neoplastic tissues [14] and plays an important role in many tumor hallmarks, including immune escape and angiogenesis, favoring tumor progression [15]. In gliomas, Gal-1 expression increases with the tumor’s grade, correlating with worse outcomes, although its specific role in GBM progression has not been fully elucidated [16,17]. Still, Gal-1 is known to play an important role in GBM immune escape, invasion, and angiogenesis [18,19,20,21].

The aim of our study has been to analyze the clinical relevance of Gal-1 expression as a prognostic factor using the GLIOCAT cohort, a homogeneously treated and the largest GBM cohort ever studied for this protein. Our previous studies, using this cohort by RNA sequencing (RNA-seq) analysis and IHC validation, led to identifying several proteins differentially expressed across different GBM molecular subtypes [11,22]. Thus, we have also analyzed the possible link between Gal-1 and these molecular markers, focusing our attention on traits of GBM like immune suppression. These analyses will help to strengthen the key role of Gal-1 in the progression and negative outcome of this aggressive disease, supporting its use as a negative prognostic biomarker for GBM patients.

## 2. Materials and Methods

### 2.1. Patients

GLIOCAT is a retrospective multicenter study including 415 consecutive patients from 6 institutions with a diagnosis of GBM (considering the recent 2021 WHO CNS tumor classification), of which 263 and 118 cases had enough tissue for IHC and molecular studies (including *MGMT* methylation and RNA-seq analysis, respectively) [22]. The clinical characteristics of these patients are summarized in Table S1. Collected samples also include 10 additional *IDH-1* mutated patients (astrocytoma grade 4 *IDH-1* mutant tumors according to the 2021 WHO classification), which were only used for the analysis of Gal-1 levels according to *IDH-1* status. All patients had been treated with the standard first-line treatment (surgery followed by radiotherapy with concurrent and adjuvant temozolomide). The cases were diagnosed and reviewed by expert neuropathologists. An independent public glioblastoma database (TCGA) was used for validation of immune cell type deconvolution (https://portal.gdc.cancer.gov/ accessed on 28 March 2018).

### 2.2. Ethical Details

This study was approved by the Institutional Review Board of the Hospital Germans Trias i Pujol (PI-14-016) and by the Ethics Committees of all the participating institutions and their Biobanks, and was conducted in accordance with the ethical standards as laid down in the 1964 Declaration of Helsinki and its later amendments. All patients or their representatives gave their written informed consent to participate in this study.

### 2.3. Tissue Microarray Preparation

Tissue microarrays (TMAs) were constructed by choosing demonstrative areas of the tumor. Between 2 and 4 areas were selected, depending on the amount of tissue available in each case. High-necrotic areas were not selected. The TMAs were constructed using a “Veridiam tissue arrayer” (Veridiam, Inc., El Cajon, CA, USA), model VTA-100, using 1-mm-diameter needles. Once the TMAs were built, consecutive sections were made at 4 microns. Hematoxylin and eosin stains were performed in sections 1, 20, and 40 to control the existence of demonstrative areas of the tumor.

### 2.4. Immunohistochemistry Analysis

Antibodies used for IHC are detailed in the Supplementary material (Supplementary Table S2). Gal-1 IHC was performed as previously reported [23]. The rest of the antibodies were performed by automated IHC using the Ventana System (Roche Diagnostics, Ventana, Tucson, AZ, USA). Expert pathologists read and evaluated the TMAs. Markers were evaluated as follows: (a) Gal-1, SOX2, CD44, and YKL40 by means of the H-score [24], which is based on the evaluation of the percentage of positive cells (0–100) and the intensity (1–3), ranging between 0 and 300. Of note, Gal-1 cytoplasmic and nuclear expression were evaluated separately, and the statistical mean of both values was calculated for correlations. Then, a cut-off of 129.3 was used to classify tumors as “low Gal-1 expression” and “high Gal-1 expression”; (b) Ki67, P53, Olig2, Nestin, TCIRG1, PD-L1, ZNF7, MEOX2, and RUNX3 as a percentage of positive cells; (c) IDO1, UBXN7, PTEN, and YWHAG as positive or negative; (d) EGFR and P16 semi-quantitatively. In particular, EGFR was evaluated as positive when the intensity of the immunostaining was maximum (3+) and negative in the rest of the cases (2+, 1+, 0); and P16 was considered negative when the immunostaining was less than 50% and positive otherwise; (e) SHC1, B4GALT1, PAX2, PGBD1, SOX11, and WASF1 as positives or negatives with a cut-off point of 1%. Those spots with a representation of less than 50% of the tumor were marked as not evaluable. A case was not evaluable when at least half of the spots were not evaluable.

### 2.5. RNA Sequencing and GBM Molecular Subtypes

In order to study GBM molecular subtypes, RNA-seq was performed as previously described in samples with enough RNA quality to be evaluated (*n* = 118) [11]. By using molecular information available from the TCGA and IGS, tumors were classified according to their RNA profiles. Tumors classified as IGS-cluster 18 were included in the classic subtype; those classified as IGS-cluster 23 were mesenchymal ones; and those classified as IGS-cluster 9 and cluster 17 were included in the proneural subtype.

### 2.6. Cell-Type Deconvolution

RNA-seq raw counts from the GLIOCAT cohort or an independent TCGA GBM cohort were normalized with the TMM method [25], and the expression data (log2CPM) was used for cell type deconvolution by CIBERSORT using the LM22 human immune signature and the CIBERSORT ‘absolute’ mode [26]. Each of the 22 deconvoluted immune cell types was correlated with the *LGALS1* RNA-seq gene expression using the ‘rcorr’ function of the R Hmisc library (extracting the Pearson’s r and asymptotic *p* values).

### 2.7. Statistical Analysis

To estimate a cutoff point for the Gal-1 H-Score, we used maximally selected rank statistics using the maxstat package [27,28] and the web-based tool Cutoff Finder [29]. Cox regression analyses were used to analyze the effects of Gal-1 on survival and identify risk factors, adjusting Gal-1 expression by age and gender. Results were shown in terms of hazard ratios. The proportional hazard assumption was checked by examining Schoenfeld residuals (for the overall model and variable by variable). The age was added as categorical, instead of continuous, to avoid the violation of this proportional hazard assumption. The Gal-1 expression relationship with clinical and molecular data, as well as the comparison between GBM molecular subtypes, were studied with the Mann-Whitney U test. Multigroup comparisons were performed with the Kruskal–Wallis test. The correlations between markers were derived from Spearman’s rho. All analyses considered *p* values < 0.05 to be statistically significant.

## 3. Results

### 3.1. Gal-1 Has Prognostic Value in GBM

**Figure 1:**
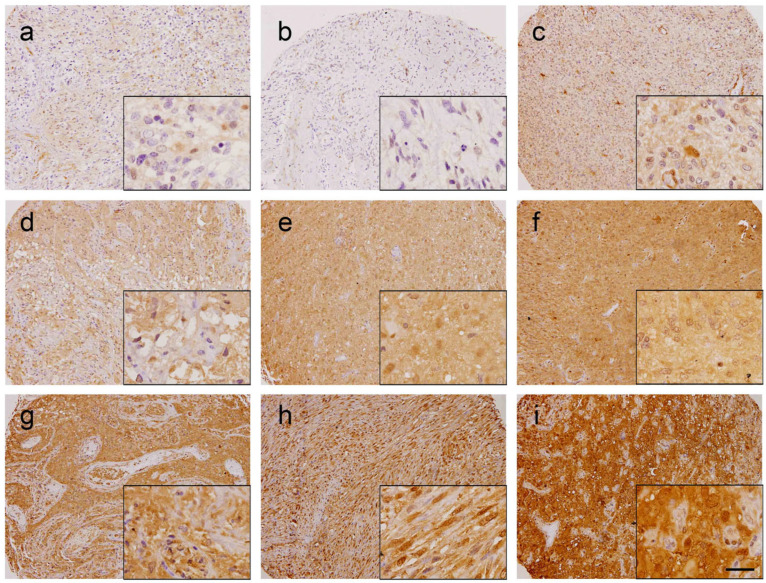
Gal-1 immunohistochemistry in glioblastoma tissue microarray (TMA). Representative images of selected “low Gal-1” (panels (**a**–**c**), H-Score < 129.3) or “high Gal-1” (panels (**d**–**i**), H-Score ≥ 129.3) glioblastoma cores are shown. Scale bar, 100 µm (low magnification) or 25 µm (high magnification inserts).

In order to analyze whether Gal-1 could have a prognostic value in GBM, 263 cases included in the TMA were evaluated for Gal-1 expression by IHC (Figure 1). Only tumor cells (identified by atypical nuclei) were evaluated for Gal-1 expression. Since Gal-1 can play different functions depending on its subcellular localization [12], both cytoplasmic and nuclear H-score staining were analyzed separately. However, cytoplasmic or nuclear Gal-1 expression did not significantly correlate with prognosis, thus the mean of the two values was calculated and used for the study. Maximally selected rank defined a cut-off of H-score at 129.3, which allowed us to classify tumors as “low” (*n* = 203) (Figure 1a–c) or “high” (*n* = 60) (Figure 1d–i) regarding Gal-1 mean expression.

**Figure 2:**
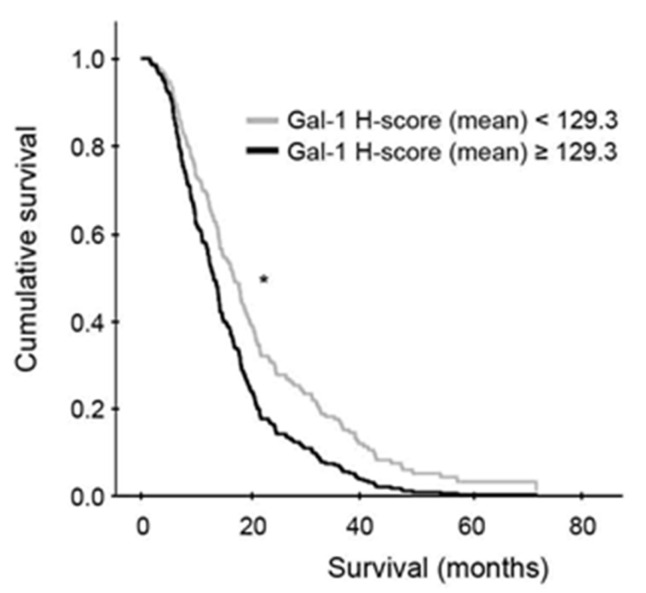
Overall survival in high- vs. low-Gal-1 mean H-scores. Kaplan-Meier curve depicting overall survival from patients with low or high mean Gal-1 H-score (*n* = 203 and *n* = 60, respectively), adjusted by age and gender through a Cox regression model. * *p* (log-rank test) < 0.05.

Remarkably, a significant correlation was observed between high Gal-1 mean H-score and worse overall survival, as patients with high Gal-1 mean H-score had a median overall survival of 12.45 months (95% CI 10.25–14.65, *n* = 60) versus 16.69 months (95% CI 14.17–19.25, *n* = 203) for those with low Gal-1 mean H-score (*p* = 0.010, Cox regression model) (Figure 2 and Table 1).

### 3.2. Correlation between Gal-1 and Clinical or Molecular Data

**Figure 3:**
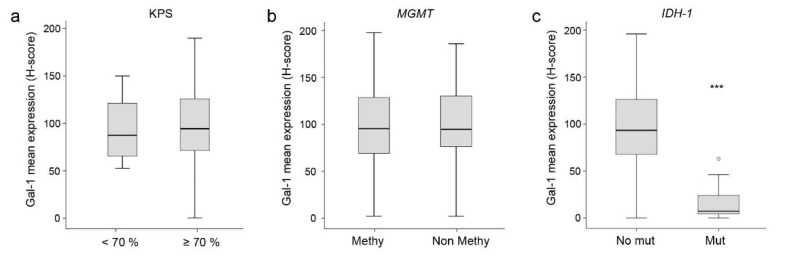
Correlation between mean Gal-1 expression and Karnofsky Performance Scale (KPS) (**a**), *MGMT* promoter methylation (**b**), or *IDH-1* status (**c**). *** *p* (Mann-Whitney U test) < 0.001.

Tumor Gal-1 H-score values were analyzed to explore the possible correlation of Gal-1 with the patient’s clinical variables. Gal-1 levels did not show any correlation with KPS or *MGMT*-methylation status (Table 2 and Figure 3a,b). We also analyzed Gal-1 levels in tumors harboring *IDH-1* mutations, a group of patients with a good prognosis who are currently classified as having an astrocytoma grade 4. Interestingly, we found significantly less Gal-1 expression in this tumor type compared to GBM (*p* < 0.001, Mann-Whitney U test) (Table 2 and Figure 3c), in line with the previously observed negative correlation between Gal-1 levels and patient prognosis in GBM.

### 3.3. Correlation between Gal-1 and GBM Molecular Subtypes

**Figure 4:**
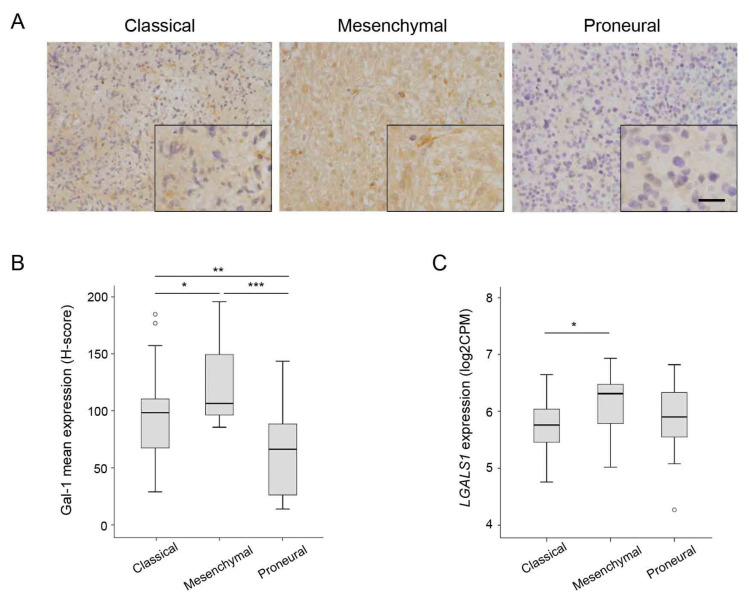
Gal-1 expression in glioblastoma molecular subtypes. (**A**) Representative images of Gal-1 immunostainings in classical, mesenchymal, and proneural glioblastoma tumors. Scale bars are 50 µm (low magnification) or 25 µm (high magnification inserts). (**B**) H-score quantification of Gal-1 protein expression depending on the molecular subtype. (**C**) *LGALS1* RNA expression (log2CPM) according to the molecular subtype. * *p* < 0.05; ** *p* < 0.01; *** *p* < 0.001 (Mann-Whitney U test).

Recent advances in genomic studies have allowed a novel classification and grading of GBM based on more accurate transcriptional biomarkers. This has resulted in the GBM molecular subgroup classification, paving the way to precision medicine. Tumors included in the GLIOCAT multicenter study were analyzed by RNA-seq and classified into the previously established GBM molecular subgroups [22]. The Gal-1 mean expression was analyzed across the different GBM molecular subtypes, with significant differences observed among them. Interestingly, Gal-1 expression was lower in the proneural group compared to the classical and mesenchymal subtypes (*p* < 0.001) (Table 3 and Figure 4A,B). A similar pattern of expression among different GBM molecular subtypes was observed when analyzing *LGALS1* RNA expression in the same samples (Figure 4C).

In the same GBM tissue samples, IHC expression of other molecular markers previously identified as being differentially expressed among different GBM molecular subtypes and related to inflammation, tumor proliferation, and angiogenesis were also evaluated [11]. Across these biomarkers, SHC1, PD-L1, PAX2, MEOX2, YKL-40, TCIRG1, and YWHAG showed a statistically significant positive correlation with Gal-1 mean expression, while OLIG2, SOX2, Ki-67, and SOX11 negatively correlated with mean Gal-1 levels (Table 4). No statistically significant correlation was detected between Gal-1 expression and other markers, such as CD44, B4GALT1, Nestin, PTEN, P16, IDO1, EGFR, p53, ZNF7, WASF1, UBXN7, RUNX3, or PGBD1 (*p* > 0.05).

### 3.4. Correlation between Gal-1 Expression and Markers of Inflammation

**Figure 5:**
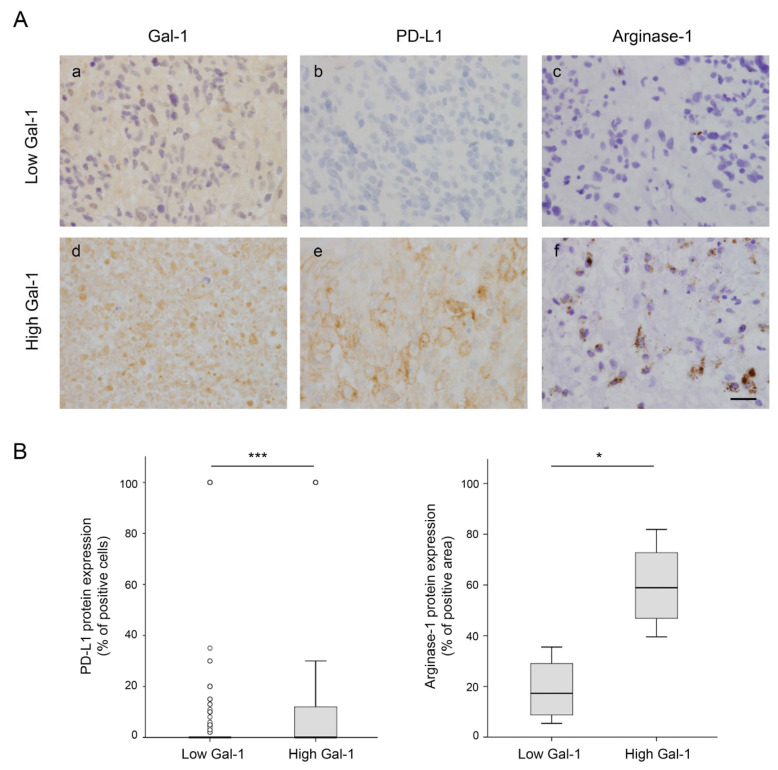
Gal-1 expression and correlation with PD-L1 or Arginase-1 expression in GBM. (**A**) Representative images of Gal-1 (**a**,**d**), PD-L1 (**b**,**e**), and Arginase-1 (labeling M2 macrophages) (**c**,**f**) immunostaining in two representative patients with low (upper lane) or high (lower lane) Gal-1 H-scores. Scale bars, 25 µm. (**B**) Immunohistochemistry quantification of PD-L1 (left panel) in GBM patients included in the TMA (*n* = 263) and Arginase-1 (right panel) in low or high Gal-1 representative patients (*n* = 8). * *p* < 0.05; *** *p* < 0.001 (Mann-Whitney U test).

Considering the role of Gal-1 in promoting an immunosuppressive environment and the importance of not only the presence of immune cell infiltrates but also their activation and effector state, we analyzed in more depth the role of Gal-1 in the modulation of the immune tumor microenvironment. Cell type deconvolution was applied to the RNA-seq data from 118 GBM patients to read the immunologic profile of the tumors and analyze its possible relation to *LGALS1* expression levels. Using the CIBERSORT computational method (Table 5), we found that *LGALS1* statistically significantly positively correlates with tumors highly enriched in M2-polarized macrophages, monocytes, and resting memory CD4-T cells, suggesting a direct association between Gal-1 expression and immune suppression. In contrast, there was a significant negative correlation between *LGALS1* and naïve CD4 T cells. The correlation between Gal-1 expression and immune evasion was also demonstrated at the protein level by IHC in tumor samples classified as low (Figure 5a) or high (Figure 5d) in terms of Gal-1 mean expression in the TMA. Tumors with high Gal-1 show increased levels of PD-L1 (Figure 5e) and Arginase-1 (a marker of M2 macrophages) (Figure 5f), compared to tumors with low Gal-1 (Figure 5b,c). These results indicate that Gal-1 expression in GBM induces tumor immune evasion by expressing the PD-L1 immune checkpoint to inhibit T cells and by promoting M2 macrophage polarization.

**Figure 6:**
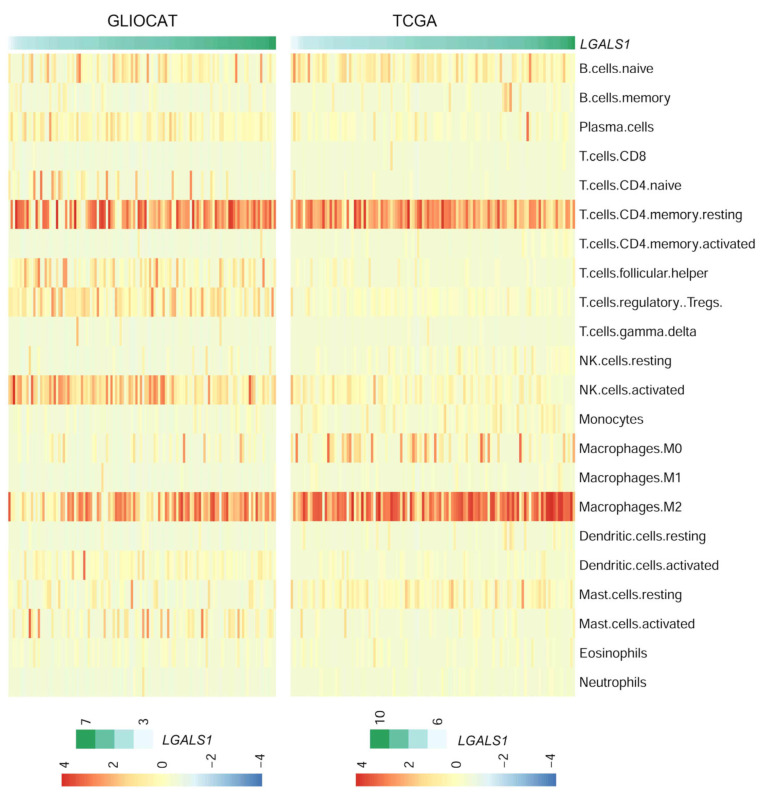
Cell type CIBERSORT deconvolution in GLIOCAT and TCGA cohorts. Heatmap showing CIBERSORT (scaled) absolute values of the LM22 immune signature for the GLIOCAT and TCGA cohorts. Samples (columns) are ordered by increasing *LGALS1* expression.

Moreover, we also validated our GLIOCAT results on immune cell type deconvolution by CIBERSORT using an independent TCGA public cohort that comprises 117 GBM tumors (Figure 6). Importantly, the immune signature associated with *LGALS1* RNA expression identified by CIBERSORT was very similar in the GLIOCAT and TCGA cohorts, with a strong positive correlation between Gal-1 levels and M2-polarized macrophages and resting memory CD4-T cells. These data strengthen the key role of Gal-1 expression in promoting immune escape in GBM.

## 4. Discussion

While several tumors have benefited from landmark advances in cancer treatment, like targeted therapies or immune therapies, these approaches have not succeeded for GBM, which remains one of the most aggressive and fatal human tumors [1,2]. A better characterization of its inter- and intra-tumor heterogeneity and a deeper understanding of its molecular biology are required to identify novel opportunities for clinical interventions.

Gal-1 is a key molecule promoting cancer progression [12,30]. This protein is overexpressed in GBM, and several reports have shown that Gal-1 downregulation impairs tumor cell growth, angiogenesis and invasion [21]. Moreover, Gal-1 triggers immune evasion in GBM by suppressing NK cells and increasing inhibitory cytokine production by M2 macrophages and myeloid-derived suppressor cells (MDSCs) [19,31]. Considering all these Gal-1 pro-tumoral functions in GBM, it is not surprising that Gal-1 expression levels correlate with poor patient prognosis [16,17]. However, these previous studies used a limited number of patients. In particular, Camby et al., reported a negative correlation between high Gal-1 levels and survival, using 41 patients with high-grade astrocytic tumors (26 with GBM), but no information about treatment was indicated. More recently, Chou et al., showed that Gal-1 overexpression was associated with short progression times and low survival in 45 GBM patients after radiotherapy alone. One of the strengths of our work is the use of a large and homogenously treated cohort of 432 GBM patients who received the standard first-line therapy (surgery followed by radiotherapy plus concurrent and adjuvant temozolomide), from which we were able to analyze 263 samples by IHC and 118 by RNA-seq. Therefore, these analyses represent a valuable piece of information in comparison to previous studies. The prognostic value of Gal-1 expression was analyzed in our cohort by H-score after IHC. High H-score expression levels of Gal-1 were significantly associated with poor prognosis in the large GLIOCAT study cohort, highlighting the negative prognostic role of Gal-1 expression.

A distinctive trait of our study is the use of the recent classification of tumors according to WHO 2021, which excludes *IDH-1* mutant patients from GBM and categorizes them as grade 4 astrocytomas. Considering the strong negative correlation between Gal-1 and *IDH-1* mutated status and the well-reported better prognosis of this subset of patients, this variable could represent a confounding factor in establishing Gal-1 as a prognostic marker in previous reports. Importantly, we found a significant correlation between Gal-1 levels and survival even after excluding *IDH-1* mutated cases, strengthening the role of Gal-1 as a prognostic biomarker. Another specific trait of our work is the analysis of Gal-1, specifically in the cytoplasm and nucleus of glioma cells. Although Gal-1 does not have a signal peptide driving cell secretion, this protein can be localized outside and inside cells, playing different roles [12,32]. For instance, through its glycan recognition ability, Gal-1 binds to several extracellular matrix components, regulating ECM organization as well as membrane glycoproteins, activating signaling pathways, and promoting migration, invasion, and metastasis. At the cytosolic level, this lectin binds to H-Ras proteins in a glycan-independent manner, stabilizing H-Ras at the cell membrane and triggering transformation [12]. Recent data has also found that acidic extracellular microenvironments, such as those in cancer, may cause Gal-1 accumulation in the nuclei of cells, where it can regulate gene expression by interaction with the transcription factor FoxP3 [33] or with Gemin-4, which is involved in RNA splicing and transport [34]. Altogether, these data indicate that Gal-1 location is associated with specific cellular functions, indicating the need to explore this also in a cancer context. Notwithstanding, our separate analysis of nuclear and cytoplasmic Gal-1 expression in GBM did not reach significant differences, suggesting that both locations are involved in Gal-1 cellular functions in this tumor.

Previous reports analyzing public datasets of RNA-seq such as the TCGA, CGGA, and Rembrandt databases indicate that Gal-1 expression is significantly higher in mesenchymal and classic subtypes compared with proneural subtypes [19]. Here, using RNA-seq data from our GBM tissue samples and excluding astrocytoma grade 4 *IDH-1* mutant tumors, we found lower expression levels of Gal-1 in the proneural molecular subtype, validating the virtual analysis performed with public databases. The same results were obtained by analyzing cytoplasmic and nuclear Gal-1 stainings independently. These data are reinforced by the fact that we observed a positive correlation between Gal-1 expression and mesenchymal markers such as YKL40, SHC1, and TCIRG1 [22], and an inverse correlation with proneural subtype markers such as Ki67, OLIG2, SOX2, and SOX11 [11,35,36,37]. Interestingly, GBM-proneural subtype patients have better prognoses, which can explain, at least in part, the increase in overall survival of patients with low Gal-1 levels found in our Kaplan-Meier analysis. In the same line, we also found significantly lower levels of Gal-1 in astrocytoma grade 4 *IDH-1* mutant tumors, which had been previously described as a positive prognostic value in GBM and currently means, even in the presence of the classical histological hallmarks of GBM, a new entity in the new 2021 WHO classification of CNS tumors [5].

Recently, a lot of interest has been centered around the role of Gal-1 in controlling tumor immune surveillance by regulating immune escape [19,31,38]. Interestingly, our RNA-seq data using CIBERSORT deconvolution analysis found a positive correlation between tumors with Gal-1 expression and tumors enriched in M2 macrophages, resting CD4 memory T cells, and monocytes, and a negative correlation between Gal-1 and CD4 naïve T cells. Our results are in concordance with data reported by Carrato et al. [11], demonstrating that mesenchymal tumors—which we here show are enriched in Gal-1—are enriched in M2 macrophages, resting memory CD4+ T cells, and dendritic cells. Our deconvolution analysis also fits with previous literature showing that Gal-1 induces migration in monocytes and shifts macrophage differentiation into a pro-resolving and anti-inflammatory M2 phenotype [38,39,40,41,42]. Moreover, previous preclinical studies demonstrated that silencing glioma tumor cells-derived Gal-1 significantly decreased the number of brain-infiltrating macrophages, Tregs, and MDSC while increasing CD4+ and CD8+ T cells [19,38]. All these articles and our work support the pivotal role of glioma-derived Gal-1 in the regulation of macrophages and myeloid cell accumulation within the glioma microenvironment. In this sense, we have also found a direct correlation between Gal-1 expression and markers linked to an inflammatory response such as TCIRG1, YKL-40, and PD-L1. Interestingly, TCIRG1 is a T-cell immunoregulator, being essential in T-cell activation and differentiation, and its expression is increased in GBM, where it is used as an indicator of lymphocyte infiltration [35] YKL40 is associated with inflammation, proliferation, and angiogenesis [43,44,45]. It is also a T-lymphocyte activator via activation of TH2 cytokine secretion in other diseases [46]. The direct relationship between Gal-1 and PD-L1 observed in this work may reflect the exhaustion phenotype of T-cells, favoring an immune suppressive microenvironment. Interestingly, in a previous article, our group observed that mesenchymal tumors had high amounts of M2 macrophages, resting memory CD4-T lymphocytes, and activated dendritic cells [11]. These results reinforce the relationship between Gal-1 and the mesenchymal subpopulation of GBM. Thus, our data suggest an opportunity to explore therapeutic combinations based on immunotherapy strategies together with Gal-1 targeted therapies in this subpopulation of patients.

## 5. Conclusions

Our study confirms that Gal-1 expression represents an independent negative prognostic factor for GBM patients homogeneously treated with standard therapy. This is one of the largest homogenous cohorts of GBM with available tissue to evaluate Gal-1 by using IHC analysis and defining an H-Score cut-off point for biomarker evaluation. Gal-1 expression was also lower in the proneural subtype of GBM, which is characterized by a better prognosis, which can explain why patients with low Gal-1 levels have increased survival. Finally, using RNA-seq deconvolution analysis, we can also confirm that Gal-1 correlates positively with M2 macrophages, resting memory CD4 cells, and monocytes, reinforcing the immune-suppressive role of Gal-1. Altogether, our study strengthens the key role of Gal-1 in GBM aggressiveness and supports its use as a negative prognostic factor and as an immune evasion biomarker.

## Figures and Tables

**Table 1:** Cox regression analyses to identify risk factors for patient survival. Results are shown in terms of hazard ratios (HR), confidence intervals (CI) at 95%, and *p* values upon Cox regression analyses.

	HR	CI 95%	*p* Value
Gal-1 mean H-score			
<129.3	1		
≥129.3	1.52	1.10–2.08	0.010
Age			
<70	1		
≥70	1.45	1.06–1.98	0.019
Gender			
Male	1		
Female	0.90	0.69–1.17	0.428

**Table 2:** Correlation between Gal-1 mean expression and relevant clinical and molecular data (Karnofsky Performance Status (KPS), O(6)-methylguanine-DNA methyltransferase (*MGMT*) status, isocitrate dehydrogenase 1 (*IDH-1*).

Clinical and Molecular Variables	*n*	Median Gal-1	Percentiles (P25–P75)	*p* Value *
KPS				
<70%	23	87.38	(62.50–128.40)	0.495
≥70%	192	94.31	(71.30–126.03)	
*MGMT* status				
Methylated	123	93.63	(66.88–127.92)	0.503
Non-methylated	133	92.82	(71.89–129.17)	
*IDH-1*				
No mutated	263	93.13	(68.44–126.25)	<0.001
Mutated	10	7.28	(3.68–29.57)	

* *p* determined by Mann-Whitney U test analysis.

**Table 3:** Correlation between Gal-1 expression and GBM molecular subtypes using the combined H-score.

	Classical	Mesenchymal	Proneural	*p* Value *
*n*	36	13	22	
Gal-1 median (P25-P75)	98.60 (67.69–111.15)	106.67 (93.28–151.46)	66.58 (26.32–90.42)	<0.001

* *p* determined by the Kruskal–Wallis test.

**Table 4:** Correlation between Gal-1 mean H-score expression and markers related to molecular subtypes.

Gal-1 Mean	Spearman Correlation Coefficient	*p* Value *
SHC1	0.230	<0.001
PD-L1	0.239	<0.001
PAX2	0.155	0.014
MEOX2	0.159	0.012
YKL-40	0.182	0.004
TCIRG1	0.151	0.017
YWHAG	0.232	<0.001
OLIG2	−0.313	<0.001
SOX2	−0.192	0.003
Ki-67	−0.283	<0.001
SOX11	−0.278	<0.001

* *p* and correlation coefficient were determined by Spearman’s rank.

**Table 5:** Correlation (Pearson’s r) between *LGALS1* expression and the CIBERSORT LM22 immune signature. *LGALS1* RNA-seq gene expression levels were correlated with each of the 22 immune cell types generated by CIBERSORT deconvolution, showing a statistically significant positive correlation with tumors enriched in resting memory T cells, monocytes, and M2 macrophages and a statistically significant negative correlation with naïve T cells.

Immune cell Type	*LGALS1* Correlation	*p* Value *
B cells, naive	0.032	0.733
B cells, memory	−0.124	0.182
Plasma cells	0.038	0.681
T cells CD8	−0.062	0.505
T cells CD4, naive	−0.193	0.036
T cells CD4 memory, resting	0.454	<0.001
T cells CD4 memory, activated	0.105	0.255
T cells folicular, helper	−0.035	0.707
T cells regulatory	0.104	0.263
T cells gamma delta	−0.019	0.835
NK cells resting	0.116	0.212
NK cells activated	−0.150	0.105
Monocytes	0.243	0.008
Macrophages M0	0.119	0.198
Macrophages M1	0.018	0.843
Macrophages M2	0.437	<0.001
Dendritic cells, resting	0.055	0.550
Dendritic cells, activated	0.163	0.078
Mast cells, resting	0.005	0.956
Mast cells, activated	0.106	0.253
Eosinophils	0.111	0.232
Neutrophils	0.067	0.474

* *p*, determined by Pearson correlation.

## Data Availability

Data is available on request due to privacy restrictions.

## Supplementary Materials

The following supporting information can be downloaded at: https://www.mdpi.com/article/10.3390/cells12060843/s1, Supplementary Table S1: Clinical characteristics of patients with Glioblastoma (WHO 2021 guidelines) included in the GLIOCAT study; Supplementary Table S2: Antibodies used for the immunohistochemical analysis of the GBM samples from the GLIOCAT cohort.
