# Supplementary material for: Gal-1 Expression Analysis in the GLIOCAT Multicenter Study: Role as a Prognostic Factor and an Immune-Suppressive Biomarker
Source: Cells. 2023 Mar 8;12(6):843. doi: 10.3390/cells12060843 (PMC10047329; doi:10.3390/cells12060843)
Supplement: Supplementary file 1 [file cells-12-00843-s001.zip › cells-2205632-supplementary.pdf]

cells

MDPI

SUPPLEMENTARY INFORMATION TO:

Gal-1 expression analysis in the GLIOCAT multicenter study: role as prognostic factor and immunesuppressive biomarker.

Neus Martínez-Bosch [1,#], Noelia Vilariño [2,#], Francesc Alameda [1,3], Sergi Mojal [4], Montserrat Arumí-Uria [1,3], Cristina Carrato [5], Iban Aldecoa [6,7], Teresa Ribalta [6], Noemí Vidal [8], Beatriz Bellosillo [1,3], Silvia Menéndez [1], Sonia Del Barco [9], Oscar Gallego [10], Estela Pineda [11], Raquel López-Martos [5], Ainhoa Hernández [12], Carlos Mesia[13], Anna Esteve-Codina [14], Nuria de la Iglesia [15], Carme Balañá [12], María Martínez-García [16,17,*] and Pilar Navarro [1,18,19,*]

Supplementary Table S1. Clinical characteristics of patients with Glioblastoma (WHO 2021 guidelines) included in the GLIOCAT study

Patients were classified as GBM according to WHO 2021 guidelines, therefore patients with isocitrate dehydrogenase 1 (*IDH-1)* mutations were excluded. Number of patientes (*n*) and % (in brackets) are included for gender, age, Karnofsky Performance Status (KPS), O6-methylguanine-DNA methyltransferase (*MGMT*) status, tumor origin and resection type.

Characteristic	Glioblastoma (WHO 2021 guidelines) *n*=263
Gender, *n* (%)	
• Men	155 (59)
• Women	108 (41)
Age, *n* (%)	
• ≤ 65 y	200 (76)
• > 65 y	63 (24)
KPS, *n* (%)	
• ≥ 70	192 (73)
• < 70	23 (9)
• Unkown	48 (18)
MGMT status, *n* (%)	
• Methylated	123 (47)
• Unmethylated	133 (50)
• Unknown	7 (3)
Tumor origin, *n* (%)	
• Primary	258 (98)
• Secondary	5 (2)
Extent of surgery, *n* (%)	
• Biopsy	19 (7)
• Partial resection	94 (36)
• Subtotal resection	65 (25)
• Complete resection	68 (26)
• Unkown	17 (6)

cells MDPI

Supplementary Table S2. Antibodies used for the immunohistochemical analysis of the GBM samples from GLIOCAT cohort

Information of the antibodies used for immunohistochemistry analysis. Details about antibody name, commercial company, research resource identifier (RRID), antigen recovery conditions (heat-CC1: tris EDTA pH 7.8) and dilution are provided. Expected localization of the antigen is indicated: N, nuclear; C, cytoplasmic; M, membrane. Evaluation criteria used for each antibody staining is as follows: H-score; a) % POS: percentage of positivity versus total number of tumor cells; b) PO/NE: positive versus negative; c) INTENS: intensity; d) SEMIQ: Semiquantitative; e) PO/NE: positive or negative, considering negativity below 1%; f) POS (Area): percentage of positive area. For more detailed information, see Materials and Methods.

Antibody	Brand (RRID)	Clone	Antigen recovery	Dilution	Localization	Evaluation
Ki67	Roche AB_2631262	30-9	heat-CC1	1/1	N	%POS (a)
P53	Roche AB_2335971	D07	heat-CC1	1/1	N	PO/NE (b)
EGFR	Ventana AB_2617183	HER1	PR-K	1/1	M/C	INTENS (c)
IDH1	Dianova	R132	heat-CC1	1/40	N/C	PO/NE (b)
PTEN	Agilent AB_2174185	6H2.1	tris EDTA pH 9.0	1/200	N/C	PO/NE (b)
SOX2	Milipore AB_827493	SP76	heat	1/100	N	H-score
OLIG2	Abcam AB_ 944547	EP112	heat	1/100	N	%POS (a)
NEST	Abcam AB_827493	SP103	heat	1/100	C/M	%POS (a)
YKL40	Abcam AB_2040911	Polyclonal	heat	1/10	C/M	H-score
CD44	Roche	SP37	heat	1/1	C/M	H-score
P16	Ventana	EH64	heat	1/1	C/N	SEMIQ (d)
PDL1	Roche AB_2819099	SP263	heat-CC1	1/1	M	%POS (a)
IDO1	Sigma AB_1846222	Polyclonal	PT Link H	1/100	N/C	PO/NE (e)
UBXN7	Abcam	Polyclonal	PT Link H	1/200	N	PO/NE (b)
YWHAG	Sigma AB_1839237	Polyclonal	PT Link L	1/500	N	PO/NE (b)
ZNF7	Invitrogen AB_2555182	Polyclonal	PT Link L	1/25	N/C	%POS (a)
TCIRG1	Sigma AB_10795138	Polyclonal	PT Link L	1/400	M	%POS (a)

cells MDPI

Gal-1	Abcam AB_138513	EPR3206(2)	citrate 10mM pH 6	1/1000	N/C	H-score
RUNX3	Abcam AB_883789	R35-G4	PT Link L	1/200	N/C	%POS (a)
SHC1	Sigma AB_1856826	Polyclonal	PT Link L	1/100	N/C	PO/NE (e)
MEOX2	Termofisher AB_2636453	Polyclonal	FLEXPLUS HIGH	1/100	N	PO/NE (e)
B4GALT1	Sigma AB_1078254	Polyclonal	FLEXPLUS LOW	1/300	C	PO/NE (e)
PAX2	Abcam	EPR 8586	PT Link H	1/200	N	PO/NE (e)
PGBD1	Termofisher AB_2635736	Polyclonal	FLEXPLUS LOW	1/25	N	PO/NE (e)
SOX11	Abcam	CL142	FLEXPLUS HIGH	1/100	N	PO/NE (e)
WASF1	Sigma AB_1080583	Polyclonal	FLEXPLUS LOW	1/50	C	PO/NE (e)
Arginase-1	Abcam AB_289571	Monoclonal	citrate 10mM pH 6	1/100	C	%POS (Area) (f)
